# The Molecular Mechanism of Transforming Growth Factor-β Signaling for Intestinal Fibrosis: A Mini-Review

**DOI:** 10.3389/fphar.2019.00162

**Published:** 2019-02-27

**Authors:** Sun-Mi Yun, Seok-Ho Kim, Eun-Hee Kim

**Affiliations:** College of Pharmacy and Institute of Pharmaceutical Sciences, CHA University, Seongnam, South Korea

**Keywords:** transforming growth factor-β, inflammatory bowel disease, intestinal fibrosis, epithelial-to-mesenchymal transition, endothelial-to-mesenchymal transition, extracellular matrix

## Abstract

Inflammatory bowel disease is known as the most chronic inflammatory disorder in colon, which subsequently progresses to intestinal obstruction and fistula formation. Many studies to date for the treatment of IBD have been focused on inflammation. However, most of the anti-inflammatory agents do not have anti-fibrotic effects and could not relieve intestinal stricture in IBD patients. Because preventing or reversing intestinal fibrosis in IBD is a major therapeutic target, we analyzed the papers focusing on TGF-β signaling in intestinal fibrosis. TGF-β is a good candidate to treat the intestinal fibrosis in IBD which involves TGF-β signaling pathway, EMT, EndMT, ECM, and other regulators. Understanding the mechanism involved in TGF-β signaling will contribute to the treatment and diagnosis of intestinal fibrosis occurring in IBD as well as the understanding of the molecular mechanisms underlying the pathogenesis.

## Introduction

Intestinal fibrosis is an unsolved issue in IBD, such as UC, and CD. An acute inflammation can be recovered from quickly, but chronic inflammation due to severe and repetitive tissue injury will progress to irreversible fibrosis with a poor regenerative capacity. It gives rise to accumulation of excessive connective tissues, eventually resulting in further complications including epilepsy, kidney disease, idiopathic pulmonary fibrosis, and heart failure. Until recently, researchers have been focusing on studying the mechanisms of inflammation to alleviate and to inhibit intestinal fibrosis. However, anti-inflammatory agents had various problems and limitations to relieve or to treat fibrosis in IBD. Therefore, in order to treat fibrotic diseases, it should no longer be focused only on inflammation and should explore the new approaches to anti-fibrotic mechanisms.

Transforming growth factor-β has a critical role in cellular responses, such as development, proliferation and differentiation. TGF-β is the master regulator to drive fibrosis in all organs including the intestine (Clouthier et al., 1997; Sime et al., 1997; Pucilowska et al., 2000; Sato et al., 2003; Rieder and Fiocchi, 2008, 2009; Wynn, 2008; Fiocchi and Lund, 2011; Mifflin et al., 2011; Speca et al., 2012). Canonical TGF-β signaling via Smads has a central role in the progression of fibrosis (Roberts et al., 2003). Nevertheless, the mechanism of TGF-β signaling in intestinal fibrosis is still not fully understood.

In this review, we analyzed clinical and therapeutic limitations for treatment of intestinal fibrosis and focused on anti-fibrotic mechanisms rather than on anti-inflammation, particularly investigating the molecular mechanism of TGF-β signaling in fibrosis occurring in IBD. Comprehension of the downstream mediators which regulate the TGF-β signaling pathway will contribute to the development of diagnostic and therapeutic agents for intestinal fibrosis in IBD.

## Clinical and Therapeutic Problems in Intestinal Fibrosis

Approximately 50% of CD patients suffer from fibrotic strictures and 75% of them will eventually undergo surgery (Siassi et al., 2007; Rieder and Fiocchi, 2009; Latella and Papi, 2012; Rieder et al., 2016). Interestingly, intestinal obstruction appeared in patients with extensive bowel resection as well as in CD patients, suggesting it cannot be completely cured by surgery and should consider new therapeutic strategies in addition to surgical treatment.

Various agents, such as salicylates, steroids, immunosuppressive drugs, and biological therapies have been used for the treatment of IBD. Such treatments may reduce the inflammatory symptoms, but do not relieve the fibrotic obstruction (Dignass et al., 2010; Van Assche et al., 2010; Latella et al., 2011; Caprilli et al., 2012). Moreover, some anti-inflammatory drugs may exacerbate fibrosis. 5-Aminosalicylic acid is known as an anti-inflammatory drug, but its anti-fibrotic effect has not yet been studied. Corticosteroids, effective agents in controlling inflammation, showed anti-fibrotic effects in various fibrotic diseases such as systemic sclerosis (Badea et al., 2009), idiopathic pulmonary fibrosis (Rogliani et al., 2008) and retroperitoneal fibrosis (Vaglio et al., 2007), with the exception of intestinal fibrosis (Graham et al., 1995). However, steroids can cause serious side effects when used for a long time. Immunosuppressive agents including azathioprine, 6-mercaptopurine, cyclosporine, and methotrexate have shown to have an anti-inflammatory effect in several chronic inflammatory diseases such as IBD. Some of the drugs have anti-fibrotic effect in pulmonary fibrosis (Dheda et al., 2004) and retroperitoneal fibrosis (Warnatz et al., 2005), however, have no significant effect on the treatment of intestinal fibrosis in IBD (Cosnes et al., 2011; Latella and Papi, 2012). Many of these anti-inflammatory treatments such as anti-TNF agents may have the effect of reducing inflammation, but they may not prevent or reverse fibrosis in IBD patients (Sorrentino et al., 2006). It suggests the possibility of new mechanisms regulating fibrosis different from the inflammation control mechanism. (Rieder and Fiocchi, 2009; Latella et al., 2013).

Despite the continuous efforts to find effective mechanisms and treatments, neither IBD progression nor surgical rates have significantly reduced (Cosnes et al., 2005; Spinelli et al., 2010; Luna et al., 2011). Especially, the mechanism of fibrosis in IBD is still not studied much compared to fibrosis occurring in other organs including the liver, lung, and kidney (Latella et al., 2014). Therefore, understanding the mechanism of TGF-β signaling may be key to alleviating and treating intestinal fibrosis.

## Canonical Transforming Growth Factor-β/Smad Pathway

Transforming growth factor-β signaling pathway is known to play a multifunctional role in the regulation of embryonic development, immunity, carcinogenesis, inflammation, and fibrosis. In normal conditions, TGF-β signaling maintains tissue homeostasis via the regulation of cell proliferation. On the other hand, TGF-β switches its function to accelerate the progress and the development of diseases such as cancer and fibrosis in abnormal conditions.

Transforming growth factor-β binds to betaglycan, also known as the type III TGF-β receptor, subsequently forming a heteromeric complex with TGF-β type II receptor. Binding of the ligands to type II receptors recruits and activates the type I receptor via the interaction between type II and type I receptors. The activated TGF-β receptors phosphorylate Smad2 and Smad3 heterodimer which interact with Smad4. The Smad2/3-Smad4 complexes translocate from the cytosol to the nucleus and bind to the additional DNA-binding cofactors to regulate specific TGF-β target genes such as plasminogen activator inhibitor-1, fibronectin, and collagen type I (Katuri et al., 2006; Schultze-Mosgau et al., 2006).

The expression of TGF-β and its receptors was increased in intestinal cells of patients with IBD, in particular in CD patients (Babyatsky et al., 1996). Also, overexpression of TGF-β in the colons of mice caused colonic fibrosis (Vallance et al., 2005). TGF-β2 is one of the most potent inducer of scarring and the TGF-β2 neutralizing antibody (Lerdelimumab) inhibited scarring after glaucoma surgery in rabbits (Mead et al., 2003). The overexpression of TGF-β2 might contribute to fibrosis by increasing ECM deposition. On the other hand, loss of Smad3 reduced tissue fibrosis in several organs, such as skin (Lakos et al., 2004), kidney (Inazaki et al., 2004), lung (Zhao et al., 2002), liver (Lee et al., 2016), and intestine (Zanninelli et al., 2006; Latella et al., 2009). Therefore, therapeutic agents targeting TGF-β for various fibrotic diseases have been investigated (Varga and Pasche, 2008; Hawinkels and Ten Dijke, 2011). Inhibitors of the TGF-β receptor, which are antibodies, that inhibit the binding of the ligand to the receptor, and antisense oligonucleotides that reduce the expression of TGF-β have been developed (Table 1). TGF-β signaling is negatively regulated by inhibitory Smad6 and Smad7 (Massague et al., 2005). Nevertheless, Smad7 is overexpressed in chronic inflammation and precancerous conditions in the colonic mucosa. Inhibition of Smad7 using antisense oligonucleotides decreased inflammation in an animal model of TNBS-induced colitis (Boirivant et al., 2006) and showed clinical remission in patients with CD (Monteleone et al., 2012). An oral antisense oligonucleotide of Smad7 called mongersen showed clinical effects on CD treatment. However, Monteleone and colleagues observed intestinal obstruction in the patients treated with 40 mg of mongersen (Monteleone et al., 2015; Vermeire, 2015). It might be caused by intestinal fibrosis, because TGF-β signaling promotes fibrosis by the synthesis and accumulation of collagen. Accordingly, Smad7 may not be a good remedy for treating intestinal fibrosis and further investigation of the Smad7 mechanism on the progression of intestinal fibrosis in IBD patients is needed.

**Table 1:** Therapeutic agents targeting TGF-β in various fibrotic diseases.

Type	Target (Drug)	Disease	Reference
Antibody	Anti-TGF-β1 antibody (Metelimumab)	Systemic sclerosis	Asano, 2017
	Anti-TGF-β2 antibody (Lerdelimumab)	Trabeculectomy patients	Khaw et al., 2007
	Anti-pan-TGF-β antibody (Fresolimumab)	Idiopathic pulmonary fibrosis	Trachtman et al., 2011
		Systemic sclerosis	Rice et al., 2015
		Myelofibrosis	Mascarenhas et al., 2014
	anti-αvβ6 antibody (STX-100)	Renal fibrosis	Hahm et al., 2007
Peptide inhibitor	TGF-β1 and -β3 (P144)	Diabetic renal fibrosis	Baltanas et al., 2013
RNA interference (RNAi)	siRNA targeting Smad4	Renal fibrosis	Morishita et al., 2014
	siRNA targeting CTGF	Peritoneal fibrosis	Liu et al., 2007
TβR kinase inhibitor	ALK5 (GW788388 )	Renal fibrosis	Petersen et al., 2008


## Epithelial-to-Mesenchymal Transition and Endothelial-to-Mesenchymal Transition

Epithelial-to-mesenchymal transition and EndMT lead to fibrosis. EMT is the main mechanism of development, cancer progression, and fibrosis (Kalluri and Neilson, 2003). During the process of EMT, epithelial cells changed to spindle-shape morphology, lost epithelial molecules such as E-cadherin, and gained mesenchymal molecules such as vimentin and alpha-smooth muscle actin (α-SMA). Epithelial markers and mesenchymal markers were regulated in the progression of intestinal fibrosis both *in vivo* and *in vitro* (Rousseaux and Desreumaux, 2006; Flier et al., 2010). These changes are mediated primarily by canonical TGF-β pathways including Smad3 but also by non-canonical TGF-β pathways including mitogen-activated protein kinase signaling and Wnt/β-catenin signaling (Bakin et al., 2002; Li et al., 2004; Wang D. et al., 2011). EMT has been described in many fibrotic diseases such as renal, pulmonary, and liver fibrosis (Kalluri and Neilson, 2003; Rastaldi, 2006; Willis and Borok, 2007; Zeisberg and Kalluri, 2008). Therefore, EMT-regulating genes can be the strategic target for intestinal fibrosis. Recently, peroxisome proliferator-activated receptor gamma (PPAR-γ) modulator, GED-0507-34 Levo, reduced EMT progression by reducing EMT-related genes in chronic colitis-associated fibrosis animal models (Di Gregorio et al., 2017).

Transforming growth factor-β is a critical inducer in EndMT as in EMT (van Meeteren and ten Dijke, 2012). EndMT also caused exaggerated myofibroblast accumulation and extracellular matrix production in several organs (Piera-Velazquez et al., 2011). TGF-β can induce collagen accumulation in connective tissues as well as morphological changes that produce differentiated cells and activated fibroblasts (Zeisberg et al., 2003; Lamouille et al., 2014). Endothelial-specific depletion of *Tgfr2* inhibited EndMT in regulating fibrotic responses to renal injury in mice (Xavier et al., 2015). The direct correlation between EndMT and IBD-related fibrosis has not yet been reported, whereas TGF-β and EndMT related genes including collagen I alpha 2 are reported to be abundant in the intestine of IBD (Burke et al., 2011; Sadler et al., 2013; Scharl et al., 2015). In this regard, EndMT can also contribute to intestinal fibrosis through differentiation of fibroblasts in IBD.

## Extracellular Matrix

Excessive production and deposition of ECM was induced in the inflammatory response and the intestinal fibrosis by activating myofibroblasts which are cells located between fibroblasts and smooth muscle cells (Rieder and Fiocchi, 2009; Speca et al., 2012). The myofibroblasts are implicated in wound healing and fibrosis. These cells induce the production of type I and type III collagens and the expression of α-SMA, and reduce the expression of ECM-degradative enzymes (Desmouliere and Gabbiani, 1995; Krieg et al., 2007). Many growth factors (PDGF, epidermal growth factor, insulin-like growth factors, and CTGF) and cytokines (IL-1 and IL-13) including TGF-β stimulate ECM synthesis through local fibroblasts leading to fibrosis (Barrientos et al., 2008). Particularly, the expression of CTGF regulated by TGF-β contributed to the progression of fibrosis (Grotendorst, 1997). Smooth muscle cells were differentiated into myofibroblasts in the condition of chronic inflammation or fibrosis (Rieder and Fiocchi, 2008, 2009). These cells actively accelerate fibrosis in IBD by inducing the production of collagen and matrix metalloproteinases (MMPs) due to stimulation of inflammatory mediators such as TGF-β. MMPs play a role in cell migration and invasion by ECM degradation in the immune response and fibrotic response as well as in physiologic function of normal cells. Therefore, regulatory factors to control ECM were focused as a therapeutic target in intestinal fibrogenesis (Luna et al., 2011). Holvoet and colleagues (2017) showed that inhibiting Rho kinases activity by administration of AMA0825 prevented and resolved intestinal fibrosis in experimental murine models and CD patient samples through inhibition of myofibroblast accumulation, expression of pro-fibrotic factors, and accumulation of ECM. In addition, Rho kinases inhibition reversed the established fibrosis in a chronic animal model and obstructed *ex vivo* pro-fibrotic protein secretion from stenotic CD biopsies (Holvoet et al., 2017). Although AMA0825 treatment did not have anti-inflammatory effects, combining AMA0825 with anti-TNF antibody in the adoptive T-cell transfer model for intestinal fibrosis could not only prevent the accumulation of fibrotic tissues but could also ameliorate inflammation. Therefore, AMA0825 may be highly valued as an additional therapeutic agent for existing anti-inflammatory drugs for CD.

## Miscellaneous

The coagulation response appears at the early stage of the wound healing mechanism which corresponds to acute inflammation. Activated platelets release growth factors including PDGF and TGF-β1, which stimulate ECM synthesis by local fibroblasts (Barrientos et al., 2008). Some publications have reported that PDGF is implicated in pulmonary, renal, and hepatic fibrosis. However, a role of PDGF in intestinal fibrosis is still unclear (Valatas et al., 2017). The thrombospondin1 (TSP1), the first discovered in the α-granules released after platelet activation, plays an important role in tissue repair (Murphy-Ullrich and Mosher, 1985). Interestingly, TSP1 is well known to activate the latent form of TGF-β1 (Lawler, 2000; Sweetwyne and Murphy-Ullrich, 2012). Treatment with intraperitoneal injections of a peptide which blocks TSP1 binding and TSP1-dependent TGF-β1 activation reduced cardiac fibrosis (Belmadani et al., 2007). Moreover, several studies have shown that treatment with TSP1 antagonist peptides can prevent bleomycin-induced lung fibrosis in mice and reduce the activation of TGF-β (Yehualaeshet et al., 2000; Chen et al., 2009; Ezzie et al., 2011). Treatment with DSS induced a more severe colitis in TSP1-/- mice, however, ABT-510, a peptide derived from TSP1 consistently diminished angiogenesis and bleeding by DSS challenge (Punekar et al., 2008). Although there is no report that TSP1 is directly involved in intestinal fibrosis, these reports suggest that TSP1 may be an attractive therapeutic target for intestinal fibrosis.

Macrophage induces TGF-β1 production in the early wound-healing response. Therefore, differentiated macrophages increased the production of PPAR-α and PPAR-γ. Recent study reported that macrophages regulate inflammation and fibrogenesis in IBD. Particularly, PPAR-γ is not only associated with adipogenesis but also with inflammation control (Rousseaux and Desreumaux, 2006). The activation of PPAR-γ with the interaction of its ligands inhibits TGF-β/Smad3 pathway by antagonizing Smad3 and by reducing CTGF expression (Zhang et al., 2009). PPAR-γ agonists attached to these receptors can prevent fibrosis induced by activated macrophages or by TGF-β1 through inhibiting fibroblast migration and proliferation (Ricote et al., 1998; Odegaard et al., 2007; Kulkarni et al., 2011).

Interleukin-17 has a critical role in promoting inflammation by increasing the production of chemokines to recruit and to activate granulocytes (Maloy, 2008). It reduced myofibroblast migration and increased the expression of MMPs and collagen production. IL-17A-mediated fibrosis required TGF-β in an animal model of pulmonary fibrosis (Wilson et al., 2010). It induced the recruitment of neutrophil, resulting in the progression of tissue damage and fibrosis in the airways (Laan et al., 1999). IL-17A expression was increased in fibrotic CD tissues compared with non-fibrotic CD tissues (Biancheri et al., 2013). In addition, its expression is associated with many types of fibrosis, such as pulmonary fibrosis (Wilson et al., 2010), myocardial fibrosis (Feng et al., 2009), hepatic fibrosis (Wang L. et al., 2011), and intestinal fibrosis (Bamba et al., 2003; Biancheri et al., 2013).

Interleukin-13 plays a role as a profibrotic mediator in the development of several fibrosis such as chronic asthma (Kumar et al., 2002), lung fibrosis (Kolodsick et al., 2004; Murray et al., 2008), systemic sclerosis (Fuschiotti, 2011), skin fibrosis (Oh et al., 2011), and ulcerative colitis (Heller et al., 2002). In the progression of fibrosis, it induces the differentiation of fibroblasts to myofibroblasts and deposition of collagens (Wynn, 2007, 2008). Also, IL-13 induced the production and secretion of TGF-β via IL-13 signaling in monocytes and macrophages (Fichtner-Feigl et al., 2006, 2008). Blockade of the IL-13 signaling leads to the inhibition of TGF-β production, resulting in the suppression of collagen deposition and the reduction of fibrotic progression. IL-13 binding to the IL-13 receptor accelerated intestinal fibrosis development in TNBS-induced chronic colitis by inducing TGF-β. Mechanistically, it has been hypothesized that IL-13 induces fibrosis by activating TGF-β (Lee et al., 2001). However, according to the research, IL-13 can act as an inducer of fibrosis independently from TGF-β by directly regulating the proliferative properties of fibroblasts, epithelial cells, and smooth muscle cells (Lee et al., 2001; Kuperman et al., 2002).

One cytokine involved in TGF-β signaling is IL-1β, a potent proinflammatory mediator, which induces EMT and myofibroblast activation through a TGF-β1-mediated mechanism (Fan et al., 2001), resulting in the development of fibrosis in IBD. Another cytokine involved in TGF-β signaling is IFN-γ, a cytokine that plays a critical role in innate and adaptive immunity, activates macrophages and induces Class II major histocompatibility complex molecule expression. It inhibits the TGF-β-induced phosphorylation of Smad3 and the activation of TGF-β target genes by inducing the expression of Smad7 (Ulloa et al., 1999). Moreover, IFN-γ regulates fibrogenesis in IBD through the reduction of fibroblast proliferation and collagen synthesis in activated myofibroblasts (Gurujeyalakshmi and Giri, 1995; Leeb et al., 2003).

## Conclusion

**FIGURE 1:**
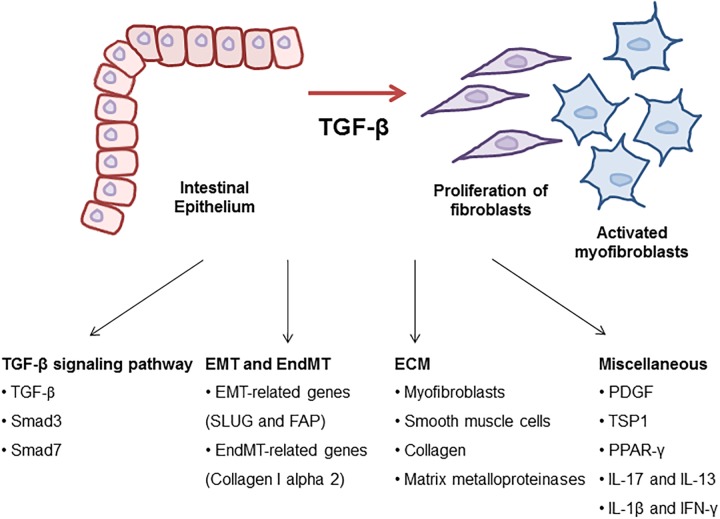
Molecular mechanism of TGF-β signaling in intestinal fibrosis. TGF-β is a key regulator to drive intestinal fibrosis occurring in IBD. In the progression of fibrosis, TGF-β signaling-related genes including TGF-β, Smad3, and Smad7 are upregulated in patients with IBD. TGF-β induces EMT and EndMT-regulating genes resulting in the progression of intestinal fibrosis. Also, ECM synthesis, including the production of collagen and MMPs, is a characteristic of intestinal strictures and luminal stenosis and is a major therapeutic challenge for IBD. Many growth factors such as TGF-β induced ECM deposition by local fibroblasts and myofibroblasts differentiated by fibroblasts. Other cytokines (IL-17, IL-13, IL-1β, and IFN-γ) and TSP1 play roles as profibrotic mediators through the activation of fibroblasts in the development of intestinal fibrosis.

The TGF-β signaling is known to be involved in the onset and the progression of many diseases such as cancer, immune diseases and fibrosis. Numerous publications have displayed that molecules related to TGF-β signaling were implicated in fibrosis (Figure 1). Many attempts have been made to develop inhibitors of TGF-β signaling pathway as a treatment. However, blockage of TGF-β1 can have high risk and problems because it is involved in other cellular functions such as differentiation, proliferation, transformation, and immune system. Additionally, the deficiency of TGF-β, Smad2, and Smad4 is lethal *in vivo* (Kulkarni et al., 1993; Nomura and Li, 1998). Despite the risk, TGF-β is still an important target in the progression of intestinal fibrosis because it correlates with the complex and diverse signaling pathways regulating the mechanism of the progression of intestinal fibrosis in IBD (Table 2). Therefore, TGF-β signaling is a potential strategy to treat and alleviate fibrosis in several fibrotic diseases including IBD (Speca et al., 2012). The challenge moving forward is to elucidate the complex mechanism of TGF-β signaling with relation to the other various signaling pathways and to find effective therapeutic agents targeting TGF-β signaling in the regulation of intestinal fibrosis in IBD.

**Table 2:** A summary of the regulators of TGF-β signaling pathway in intestinal fibrosis.

Mechanism		Target (drug)	Effects	Reference
Downregulators	Canonical TGF-β/Smad pathway	Smad3 knockout mice	Resistance to intestinal fibrosis	Zanninelli et al., 2006; Latella et al., 2009
		Antisense oligonucleotides of Smad7 (Mongersen)	Clinical remission in CD patients	Boirivant et al., 2006; Monteleone et al., 2012
	EMT	PPAR-γ modulator (GED-0507-34 Levo)	Reduced chronic colitis-associated fibrosis in animal model	Di Gregorio et al., 2017
	Cytokine	Blockade of the IL-13	Reduced TNBS-induced chronic colitis	Fichtner-Feigl et al., 2006, 2008
Upregulators	Canonical TGF-β/Smad pathway	TGF-β and its receptors	Increased in patients with IBD	Babyatsky et al., 1996
	ECM	Type I collagen alpha 2	Abundant in the intestine of IBD	Luna et al., 2011; Sadler et al., 2013
	Cytokine	IL-1β and IFN-γ	The progression of intestinal fibrosis in IBD	Leeb et al., 2003
		IL-17	Increased in fibrotic tissues with CD	Bamba et al., 2003; Biancheri et al., 2013
		IL-13	The intestinal fibrosis development in TNBS-induced mice	Fichtner-Feigl et al., 2008


## Author Contributions

S-MY prepared the manuscript. S-HK reviewed the drafts and provided important information. E-HK conceived the idea, reviewed the drafts, and provided important information for the completion of this manuscript. All authors contributed to the writing and final approval of the manuscript.

## Conflict of Interest Statement

The authors declare that the research was conducted in the absence of any commercial or financial relationships that could be construed as a potential conflict of interest.

## Abbreviations

α-SMA: alpha-smooth muscle actin
CD: Crohn’s disease
CTGF: connective tissue growth factor
DSS: dextran sulfate sodium
ECM: extracellular matrix
EMT: epithelial-to-mesenchymal transition
EndMT: endothelial-to-mesenchymal transition
IBD: inflammatory bowel disease
IFN-γ: interferon-gamma
IL: interleukin
MMPs: matrix metalloproteinases
PDGF: platelet-derived growth factor
PPAR: peroxisome proliferator-activated receptor
TGF-β: transforming growth factor-beta
TNBS: trinitrobenzene sulfonic acid
TNF: tumor necrosis factor
TSP1: thrombospondin1
UC: ulcerative colitis
